# Developmental origins of natural sound perception

**DOI:** 10.3389/fpsyg.2024.1474961

**Published:** 2024-12-11

**Authors:** Silvia Polver, Nicole Miller-Viacava, Matthieu Fraticelli, Judit Gervain, Christian Lorenzi

**Affiliations:** ^1^Department of Developmental and Social Psychology, University of Padua, Padua, Italy; ^2^Padova Neuroscience Center, University of Padua, Padua, Italy; ^3^Laboratoire des Systèmes Perceptifs, UMR CNRS 8248, Ecole Normale Supérieure, PSL University, Paris, France; ^4^Integrative Neuroscience and Cognition Center, UMR8002, Université Paris Cité and CNRS, Paris, France

**Keywords:** human auditory ecology, auditory development, infants, children, environmental sounds, natural soundscapes, animal vocalizations, water sounds

## Abstract

Infants are exposed to a myriad of sounds early in life, including caregivers' speech, songs, human-made and natural (non-anthropogenic) environmental sounds. While decades of research have established that infants have sophisticated perceptual abilities to process speech, less is known about how they perceive natural environmental sounds. This review synthesizes current findings about the perception of natural environmental sounds in the first years of life, emphasizing their role in auditory development and describing how these studies contribute to the emerging field of human auditory ecology. Some of the existing studies explore infants' responses to animal vocalizations and water sounds. Infants demonstrate an initial broad sensitivity to primate vocalizations, which narrows to human speech through experience. They also show early recognition of water sounds, with preferences for natural over artificial water sounds already at birth, indicating an evolutionary ancient sensitivity. However, this ability undergoes refinement with age and experience. The few studies available suggest that infants' auditory processing of natural sounds is complex and influenced by both genetic predispositions and exposure. Building on these existing results, this review highlights the need for ecologically valid experimental paradigms that better represent the natural auditory environments humans evolved in. Understanding how children process natural soundscapes not only deepens our understanding of auditory development but also offers practical insights for advancing environmental awareness, improving auditory interventions for children with hearing loss, and promoting wellbeing through exposure to natural sounds.

## 1 Introduction: human auditory ecology

Infants encounter a myriad of sounds early in life. Their caregivers' speech, songs heard at daycare, the family dog's barking, leaves rattling in the park, and birds singing are all part of the earliest human experiences. Decades of research have shown that young infants have sophisticated perceptual abilities to process speech, laying the foundations for language acquisition (Nallet and Gervain, 2021; Werker, 2018). Children's sensitivity to music is also beginning to be understood (Trehub and Hannon, 2006; Winkler et al., 2009; Trainor and Unrau, 2011). Much less is known about how infants perceive sounds that are not generated by humans, in particular how they perceive natural environmental sounds and soundscapes. Yet, understanding how infants process natural auditory signals is fundamental to the study of auditory development, and human development more generally (Cummings et al., 2009).

Further, this endeavor is central to the development of a new field called “human auditory ecology,” the scientific study of human beings' ability to perceive the ecological processes at work in natural habitats (Lorenzi et al., 2023). For many non-human species, being able to detect, discriminate, identify, and orient toward natural sounds such as animal vocalizations, and geophysical sounds (i.e., wind, rain or a stream of water) determines survival and reproduction through the ability to represent and monitor the immediate acoustic environment. A fundamental question of this novel field of research is, therefore, whether these auditory abilities and underlying mechanisms operate throughout the life span or whether they emerge through exposure, learning and cultural transmission. Urban habitats and spoken language are relatively recent in humanity's history and evolution. By contrast, natural soundscapes—the complex arrangements of animal vocalizations and geophysical sounds as shaped by sound propagation characteristics of natural settings such as forests or savannahs (Grinfeder et al., 2022)—have preceded the appearance of *Homo sapiens* 300,000 years ago (Senter, 2008).

**Figure 1:**
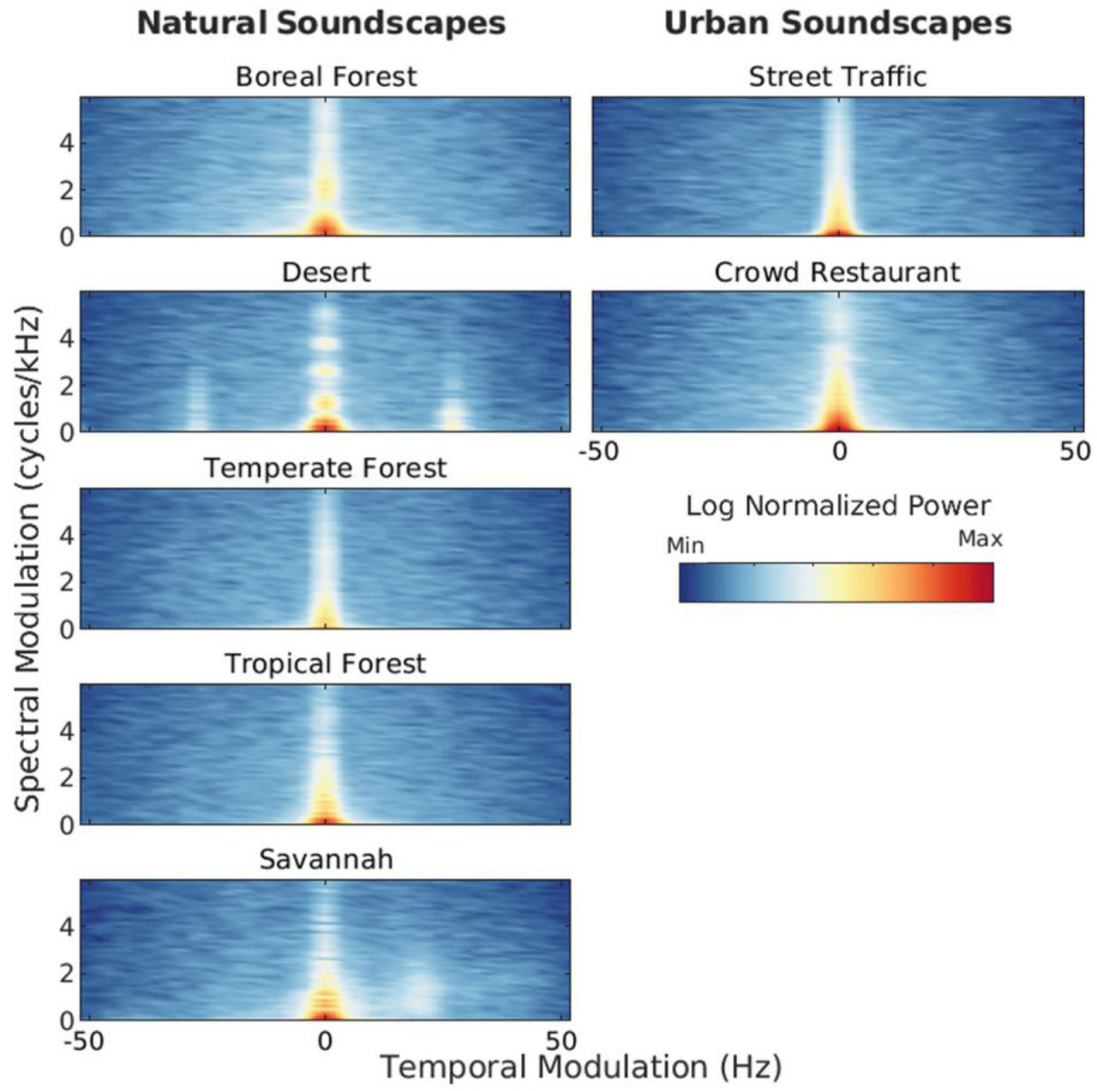
Modulation power spectra (MPS) of natural versus urban soundscapes. MPS shows how modulation power varies as a function of spectral-modulation (ordinate) and temporal-modulation (abscissa) rate (see Singh and Theunissen, 2003 for more information about MPS analysis). These representations highlight the spectral and temporal structure in the spectrogram of sounds (Theunissen and Elie, 2014). MPS were computed on single acoustic recordings conducted in closed and open terrestrial natural habitats (boreal, temperate and tropical forests, a savannah and a desert), and in two typical indoor and outdoor urban settings (street traffic and crowd). Each MPS is normalized by its own maximum modulation power. Sources: B. Krause, Wild Sanctuary (natural soundscapes); S. Meunier, LMA, CNRS, and royalty free sound library SoundBible (urban soundscapes). See Supplementary Appendix for additional information about the stimuli.

Natural and urban soundscapes differ in many ways. Figure 1 illustrates some of the spectro-temporal differences between natural soundscapes recorded in protected nature reserves (specifically, forests, savannah and desert) and common urban soundscapes (specifically, street traffic and crowd in a restaurant). Figure 1 shows the modulation power spectra of single acoustic samples of natural vs. urban soundscapes selected from our database (Singh and Theunissen, 2003, see also the Supplementary Appendix). Additional analyses (average modulation power spectra calculated over a larger corpus of acoustic samples) are presented in Supplementary Figure 6 of the Appendix. For instance, Figure 1 reveals that unlike urban soundscapes, the soundscapes recorded in the desert, tropical forest and savannah show greater modulation power for relatively fast temporal modulation and for relatively high spectral modulation, reflecting the rapid, periodic trills and harmonic structure of insect stridulations and bird vocalizations, respectively.

It is reasonable to assume that auditory mechanisms involved in the passive and active monitoring of natural sounds and soundscapes have an ancestral origin shared with many non-human species equipped with tympanic ears, predating the occurrence of spoken language and “cocktail party” situations. These ancestral mechanisms may be optimized through evolution for spectro-temporal cues quite different from those typically found in more recent urban settings. Furthermore, it is also reasonable to assume that exposure, learning and expertise shape our ability to monitor natural sounds and soundscapes. Consistent with this idea, expert listeners have been found to adopt a more analytical listening strategy prioritizing precision, whereas non experts attend to soundscapes in a more holistic way (Guastavino, 2003). It follows that developmental studies exploring infants and children's ability to perceive animal vocalizations and geophysical sounds should help build theoretically more solid foundations for human auditory ecology by clarifying the factors responsible for our ability to build a clear sense of place and time through our ears and auditory brain (Gervain and Mehler, 2010; Lickliter and Witherington, 2017; Oyama, 1979; Reh et al., 2020; Werker and Hensch, 2015).

## 2 How do children perceive environmental sounds?

**Figure 2:**
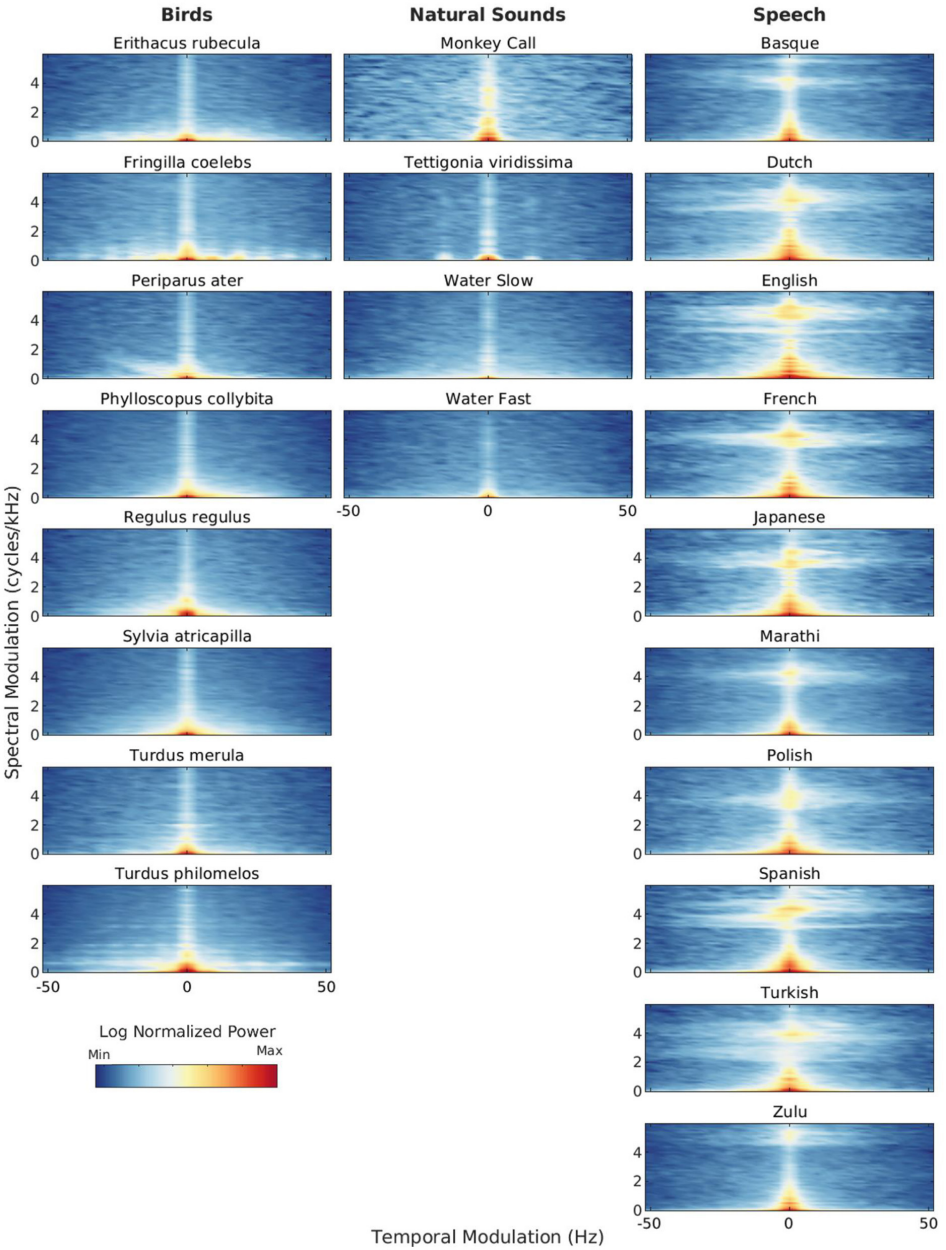
Modulation power spectra (MPS) of natural sounds (bird vocalizations, primate vocalization, insect stridulation and water sounds) and speech sounds. Natural sounds: (i) Bird songs: single recordings from eight bird species selected from a protected European cold forest in the East of France (the Risoux forest in France). Sources: J.-C. Roché & MNHN; (ii) Primate vocalization: single recording of a baboon “wahoo” vocalization. Source: Gemignani and Gervain (2024); (iii) Insect stridulation: single recording of *Tettigonia viridissima*, the great green bush-cricket inhabiting the Risoux forest. Source: J. Sueur, MNHN; (iv) Water sounds: single recordings of a single headwater forest stream with distinct water temperatures and discharge rates (here, a slow versus a fast discharge). Source: Klaus et al. (2019). Speech sounds: single sentences recorded in ten different languages from a female speaker: Basque, Dutch, English, French, Japanese, Marathi, Polish, Spanish, Turkish, and Zulu (1 recording per language). Source: Ramus et al. (1999). Each MPS is normalized by its own maximum modulation power. See Supplementary Appendix for additional information about the stimuli.

From birth, infants are exposed to many different natural sounds such as rain, thunder, rustling leaves, streams of water and birds chirping. What are the acoustic properties of these sounds? Figure 2 illustrates the spectro-temporal similarities and differences between natural sounds such as bird vocalizations, insect stridulations, primate vocalizations and streams and speech sounds from a variety of languages. Similarly to Figure 1, Figure 2 shows modulation power spectra of single acoustic samples of natural vs. speech sounds selected from our database. Additional analyses (average modulation power spectra and modulation statistics calculated over a larger corpus of acoustic samples) are presented in Supplementary Figure 7 of the Appendix. Consistent with Singh and Theunissen's (2003) canonical study, natural sounds are lowpass in shape: they show most of their modulation power for low temporal and spectral modulations. Speech sounds and to a some extent primate vocalizations and some bird songs show more spectral modulation power at relatively high spectral modulations, indicating the presence of fine-grained harmonic structure. Insect sounds do not show this spectral feature, but have more modulation power at relatively high temporal modulations due to fast, periodic stridulations/timbalations. Some bird songs also show this temporal feature, presumably caused by fast trills. Streams of water show none of these spectro-temporal features, they are more similar to broadband noise.

Does the perception of natural sounds—especially (non-human) animal sounds—rely on neural mechanisms shared with speech processing (Gervain et al., 2014; Vouloumanos et al., 2010) or are they distinct? The efficient neural coding hypothesis suggests that the mammalian sensory system evolved to encode sensory information optimally (Simoncelli and Olshausen, 2001). Thus, our perceptual systems are optimized for natural stimuli (Gervain et al., 2014), and language evolved leveraging the capabilities of these systems (Lewicki, 2002; Smith and Lewicki, 2006). This account implies that speech perception and the perception of natural sounds have shared underlying neural representations. By contrast, speech has been argued to be “special,” since it is our species-specific communicative signal, and as such a sound that our vocal tract can produce. This link with the motor system and the auditory feedback loop distinguishes speech from other sounds, which we can only perceive, but not produce (Liberman and Mattingly, 1985). Despite these theoretical debates, studies are only now starting to explore how we perceive natural sounds, in particular in their full ecological complexity, e.g. natural soundscapes (Lorenzi et al., 2023).

Investigating development is highly relevant to these theoretical questions, as the similarities and differences between the developmental trajectories of speech perception and natural sound perception abilities can shed light on whether or to what extent they share underlying mechanisms. Further, infants and young children often have limited experience of some of these sounds categories. It is thus easier to determine what auditory sensitivities are biologically endowed, possibly shaped by our evolutionary history, and which ones require experience to emerge. To date, however, only a few studies have tested the perception of natural soundscapes in developmental research, each with distinct research objectives.

### 2.1 How do children perceive animal vocalizations?

Many animal species communicate with auditory signals. Of these, two groups have received particular attention in the study of children's perceptual sensitivities: primates, our closest phylogenetic relatives, who have vocal tracts at least somewhat comparable to our, and birds, some of which produce particularly complex, elaborate, acoustically rich and at least to some extent combinatorially productive vocalizations.

Many studies have focused on how at birth and in the first months of life infants process the vocalizations of primates. Vocalizations are a salient signal from birth onward and share evolutionary significance across species (Cristia et al., 2014). Interestingly, a significant number of studies reported no selective processing for human speech compared to primate vocalizations from birth up to 3–4 months of life, despite maintaining behavioral listening preferences for vocalizations of biological origin over artificial sounds (Cristia et al., 2014; Ferry et al., 2013; Perszyk and Waxman, 2016; Vouloumanos et al., 2010; Shultz and Vouloumanos, 2010). This broad preference suggests that similar neural processes might be involved in the early perception of both human and non-human vocalizations, at least within the more general primate category (Perszyk and Waxman, 2016). Indeed, since speech and non-human primate vocalizations share certain acoustic properties such as a harmonic structure (Figure 2), given the similarities between humans and other primates' vocal tracts (Altmann et al., 2007; Smith and Lewicki, 2006), newborns may be inherently drawn to harmonically rich sounds with spectral and temporal irregularities (Belin, 2006). The same reasoning applies to the temporal structure of speech and primate vocalizations, which are both characterized by relatively slow amplitude-modulation, i.e., temporal envelope patterns reflecting neural and motor constraints on articulatory processes (Figure 2 and Supplementary Figure 7).

This broad initial sensitivity to primate vocalizations (Vouloumanos et al., 2010) may then be sharpened into more specific preferences for speech by early experience between 4 and 6 months (Scott et al., 2007; Vouloumanos et al., 2010; Perszyk and Waxman, 2016). Indeed, one study examining neural responses in 4-month-old infants found that primate vocalizations and speech activated similar brain regions. However, speech triggered stronger activity in the left hemisphere, while monkey vocalizations elicited greater activity in the right hemisphere (Minagawa-Kawai et al., 2011). This aligns with the maturation of auditory cortices in the first months of life (Polver et al., 2023). However, further studies are necessary to understand the neural underpinnings of these processes.

Some studies also looked at infants' sensitivity to bird songs. Bird vocalizations are the most frequent biotic components of natural soundscapes (Lorenzi et al., 2023) and convey salient spectro-temporal cues that make them easy to distinguish from other animal acoustic productions such as insect stridulations or primate vocalizations (Figure 2 and Supplementary Figure 7; see also Catchpole and Slater, 2008; Fay and Popper, 1994; Hoy et al., 1998 for reviews). One study investigated whether infants could behaviorally distinguish between repetitive, low-frequency sounds made by sea birds and melodious, high-frequency songs of garden birds (Lange-Küttner, 2010). The study hypothesized that infants might be more responsive to the low-frequency sea bird sounds, which fall within the frequency range of the human voice, possibly due to the relative immaturity of their auditory systems (Lange-Küttner, 2010). Infants were recruited from Aberdeen, Scotland, a harbor town where sea birds are common. The participants included 5- to 7-month-old infants, 10- to 12-month-old infants, and Scottish undergraduate students (Lange-Küttner, 2010). Infants showed a preference for sea-bird sounds, whereas adults preferred garden-bird songs. Older infants (10- to 12-month-olds) were in between, as they began to show increased preferential looking times to garden-bird songs, though they still preferred sea-bird sounds (Lange-Küttner, 2010). To determine if familiarity with sea-bird sounds influenced these results, a follow-up experiment was conducted in central Europe (Leipzig, Germany) with 4–5- and 6–8-month-old infants as well as in London with adults from diverse ethnic backgrounds. In these locations, sea birds are not part of the natural habitat. Infants still preferred sea-bird sounds, while adults preferred garden-bird songs, suggesting that the preference for sea-bird sounds in infants might be a universal disposition rather than a result of local exposure (Lange-Küttner, 2010). In this study, it was also tested whether individual bird calls influence preference within bird categories by investigating if certain exemplars have a greater impact on looking behavior. If individual exemplars strongly drive preference due to their specific acoustic characteristics, stronger within-category preferences would be observed. Conversely, if attention is evenly distributed among exemplars, indicating a representation of the category, evenly distributed preferences between seabird and garden bird categories would be expected. German infants showed fewer within-category preferences for both sea-bird sounds and garden-bird songs and more between-category preferences than Scottish infants. This suggests that the local environment may still shape universal biases, as greater exposure to sea-bird sounds might have facilitated early perceptual categorization in the Scottish infants (Lange-Küttner, 2010). These findings highlight the need for studies conducted in different settings, e.g. rural, wild or urban, to explore the effects of exposure and experience (Lorenzi et al., 2023).

Another study compared infants' responses to bird songs and speech in an unfamiliar language (Santolin et al., 2019). The study examined 4-month-olds' looking preferences for bird song (sung by a European starling) compared to sentences in Mandarin Chinese that either maintained normal prosodic features (Forward condition) or violated them (Backward condition), using an infant-controlled looking time preference procedure (Santolin et al., 2019). The findings showed that infants preferred bird songs over backward speech but did not exhibit a preference between forward speech and bird songs. This suggests that infants are drawn to naturally produced sounds, whether human or non-human, as reliable sources for learning (Santolin et al., 2019; Ravignani et al., 2019).

Currently, there are no studies, to our knowledge, that have examined the neural mechanisms of children's perception of bird song and other non-mammalian vocalizations. This underscores the need for further research in this area.

### 2.2 How do children perceive water sounds?

Studies investigating how children process non-biological natural sounds are particularly limited. Of this sound category, essentially only water sounds have received any attention so far. This is not surprising as water holds a unique significance within natural environments due to its fundamental role for survival (Lorenzi et al., 2023). Water also has unique acoustic properties (Geffen et al., 2011; Guyot et al., 2017; McDermott et al., 2009). Water sounds belong to the broad category of “textures,” that is quasi-stationary sounds resulting from the superimposition of many independent sound sources (i.e. bubbles). Sound textures are assumed to be perceived through a temporal integration process discarding acoustic details and keeping only summary statistics (McDermott et al., 2009).

**Figure 3:**
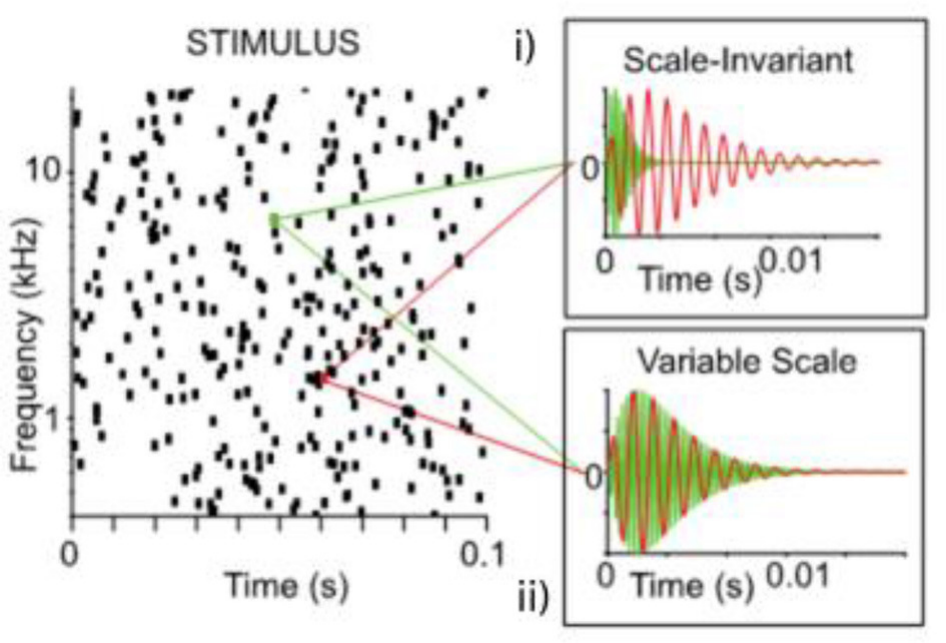
The generative model of water sounds used in Geffen et al. (2011) and Gervain et al. (2014). The model generates sounds using a population of gammatone chirps, each defined by its frequency, amplitude and cycle constant of decay. These parameters can be set such that the chirps are (i) scale invariant (upper inset), i.e. the cycle constant of decay is fixed and therefore the shape of the chirp is constant, frequency is inversely proportional to duration, or (ii) variable scale (lower inset), i.e. duration is held constant, and independent of frequency, therefore the shape of the chirp changes.

**Figure 4:**
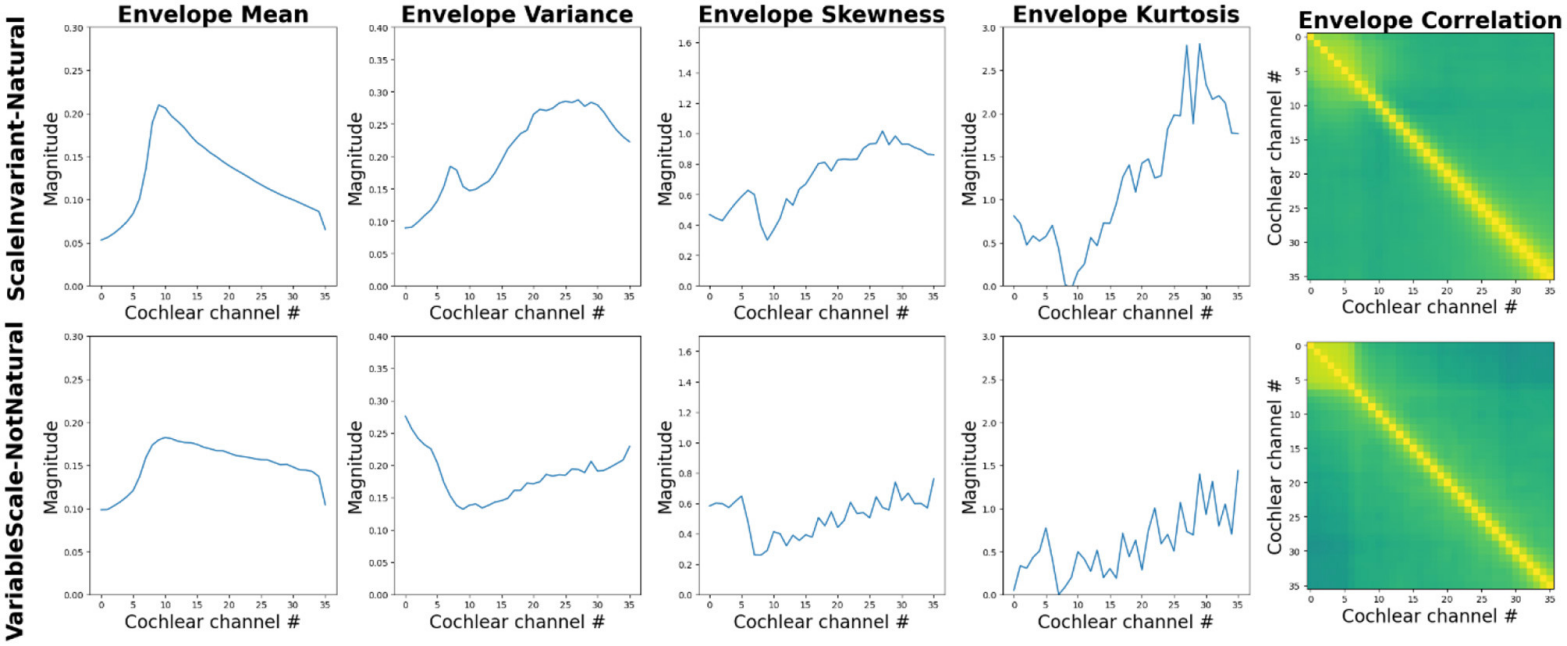
Summary statistics computed by a model of auditory texture perception (McWalter and Dau, 2017) in response to the scale-invariant (“natural,” **top panels**) and scale-variable (“not-natural,” **bottom panels**) synthetic water sounds used by Geffen et al. (2011) and Gervain et al. (2014)). Summary statistics mean, variance, skew and kurtosis (ordinate) are shown as a function of cochlear channel (abscissa). Cross-band correlations (right-most panels) are shown for each pair of cochlear channels (the hue value from green to yellow covers the 0–1 range of cross-band correlations). The two synthesized sounds differ substantially in terms of their excitation pattern (the internal power spectrum of sounds) and sparsity in each cochlear channel, with scale-invariant (natural) sounds being sparser than scale-variable (not-natural) sounds. The coordination of the temporal envelopes at the output of the cochlear channels also appears to be somewhat different between the two sounds. See Supplementary Appendix for additional information about the computational auditory model.

Water sounds are among the first auditory stimuli encountered by infants, making them inherently familiar (Gervain et al., 2014). Two studies (Gervain et al., 2014, 2016) thus investigated how water sounds are processed across development. In both studies, a generative model with a small set of parameters was used to generate sounds of running water (Geffen et al., 2011). Specifically, the parameters of the model were set in such a way that the resulting sounds were either scale-invariant, i.e. did not have any privileged temporal scale, characteristic of many natural sounds, or they were variable scale (Figure 3; Geffen et al., 2011). Figure 4 shows that texture statistics computed by a model of the human auditory system differ—sometimes substantially as in the case of statistics estimating temporal envelope sparsity—across the scale-invariant and variable-scale synthetic water sounds used by Gervain et al. (2014, 2016).

Scale-invariant sounds generated by the model were rated by human adults as natural, and described verbally as different instances of water sounds (e.g. rain, running tap etc.). When scale-invariance across spectral bands was violated, adults did not perceive the sounds as natural. Gervain et al. (2014, 2016) investigated whether very young infants were also sensitive to scale-invariance in water sounds.

The first study (Gervain et al., 2014), which focused on 5-month-old infants, habituated infants to either scale-invariant or variable-scale sounds. When habituated to scale-invariant sounds, infants looked significantly longer to a change to variable-scale sounds, whereas infants habituated to variable-scale sounds showed no such difference. These results suggest that infants were able to form a perceptual category of the scale-invariant, i.e. natural water sounds, but not of variable-scale sounds, which indeed are not perceived as natural sounds by adults either. Further, infants showed no preference between those scale-variant water sounds that adults judged more typical (e.g. rain) and those that they judged less typical, suggesting that scale-variance is possibly a more important feature of water sounds for infants than familiarity.

One aspect not investigated in these studies, as noted by the authors, is the influence of experience and initial exposure. In the second study, therefore, the same stimuli were presented to newborn infants between 0–3 days old, and fNIRS was utilized to uncover the neural mechanisms involved in processing these sounds (Gervain et al., 2016). The results revealed that newborns are able to process the statistical properties of scale-invariant natural stimuli, successfully discriminating variable-scale and scale-invariant stimuli, similarly to 5-month-olds. The localization of the differential response in the left frontal and temporal areas aligns with adult studies, which demonstrate that rapidly changing auditory events preferentially engage the left temporal areas (Hickok and Poeppel, 2007; Zatorre et al., 2002; Gervain et al., 2016). These findings indicate that the human brain is ready from early life to process natural sounds as distinctive signals (Gervain et al., 2016).

**Figure 5:**
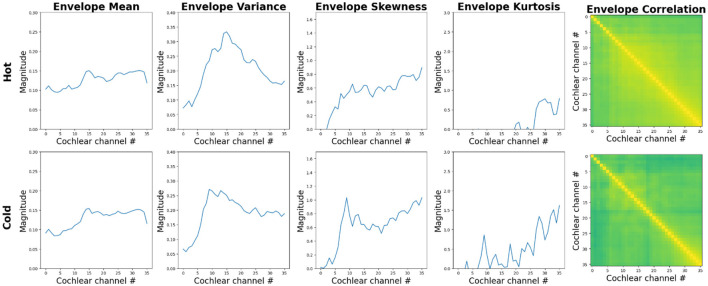
Summary statistics computed by a model of auditory texture perception (McWalter and Dau, 2017) in response to the hot **(top panels)** and cold **(bottom panels)** water sounds used by Agrawal and Schachner (2023). The two sounds show comparable power spectra and envelope sparsity in each cochlear channel. However, the coordination of the temporal envelopes at the output of the cochlear channels appears larger for the sound of hot water. See Supplementary Appendix for additional information about the stimuli and computational auditory model.

Interestingly, however, a recent study by Agrawal and Schachner (2023) suggests that children's sensitivity to a specific attribute of water sounds, temperature undergoes developmental refinement. Figure 5 shows how texture statistics, especially cross-band temporal-envelope correlations, differ between the sounds of cold and hot water, used by Agrawal and Schachner (2023). The authors found that children's ability to estimate the temperature of water from its sound, robust in adults, is not yet present in children between 3–6 years of age. This skill appears only in middle childhood at ages 7–11 years, and develops gradually over the first decade of life. These age-related differences in children may be partially driven by varying amounts of relevant experience and changes in auditory sensitivity over the course of childhood.

The perception of water sounds has also been explored in atypically developing children. Testing anecdotal reports suggesting that children with Williams syndrome have exceptional skills for recognizing environmental sounds by timbre, one study presented water sounds (e.g., sea, shower, fountain, waterfall, river) and sounds produced by walking (e.g., running downstairs, walking on shingle, walking on pavement, walking on rubble, running on pavement) to children with Williams syndrome, Down syndrome, and typically developing children in an identification task. Results showed that children with Williams syndrome performed lower than their typically developing peers and similarly to those with Down syndrome for both types of sounds. This indicates that Williams syndrome children do not have increased auditory sensitivity, challenging previous claims (Martínez-Castilla et al., 2015). Rather, both groups of atypically developing children showed poorer identification than typically developing peers.

### 2.3 How do children categorize different sounds?

Some studies approached the question of the specificity of children's auditory perception by comparing their perception of a wide variety of different sound categories. One of the first such studies, Shultz and Vouloumanos (2010) compared 3-month-old infants' listening patterns to speech in unfamiliar languages, rhesus macaque vocalizations, human non-communicative vocalizations, human non-speech communicative vocalizations, and environmental sound stimuli. Environmental sounds comprised mechanical sounds (e.g., bells, hammers) and natural geophysical sounds (e.g., wind, rain) commonly found in infants' surroundings (Shultz and Vouloumanos, 2010). The study found that 3-month-old infants listened longer to speech than to any other sound category. However, the difference was less pronounced when speech was compared to environmental sounds (Shultz and Vouloumanos, 2010). The authors suggest that infants may not perceive mixed environmental sounds as a coherent category because they originate from diverse sources (Shultz and Vouloumanos, 2010). This strongly implies a necessity for more controlled experiments in this area.

Another study focused on the development of sound-object associations using environmental sounds (e.g. animal cries, human nonverbal vocalizations, vehicle noises, alarms, water sounds, and music) and their matched verbal descriptions in 15–20-month-old infants (Cummings et al., 2009). The authors observed that toddlers were better able to learn associations for both types of sounds with age, but there was no difference between the two sound types (Cummings et al., 2009).

A more recent study investigated 6–12-year-old-children's attitudes to different soundscapes, encompassing adult conversation, children's play, nature sounds (such as leaves rustling and water sounds), animal sounds (like barking and bird songs), motorized and electromechanical sounds (such as traffic and construction), and classical music (Su et al., 2023). The findings revealed that children anticipated more social interaction when exposed to children's sounds and nature sounds, possibly due to social cues and interactive activities like water play. In contrast, they associated animal sounds and classical music with solitary activities, owing to their perceived relaxing and restorative qualities. Intriguingly, these findings resonate with reports of the restorative effects of environmental sounds observed in adults (Su et al., 2023; Lorenzi et al., 2023). Conversely, motorized and electromechanical sounds were generally avoided by children (Su et al., 2023).

### 2.4 Need of ecological approaches

The use of acoustic databases collected by soundscape ecologists and eco-acousticians (Sueur and Farina, 2015) and implementation of novel behavioral paradigms inspired by cognitive ethologists offer developmental psychologists and neuroscientists unique opportunities to set up experimental paradigms with enhanced ecological validity. In that respect, future work should consider testing infant and children with acoustic stimuli and behavioral tasks targeting the repertoire of natural auditory behaviors in humans (Kingstone et al., 2008; Miller et al., 2022), that is sounds and behaviors involved in *environmental monitoring* (Keidser et al., 2022) by contrast with communication behaviors. The objective would be to study the human capacity to process natural soundscape information in (truly) ecologically-valid situations (Lewkowicz, 2001; Schmuckler, 2001; Holleman et al., 2020), that is for stimuli and tasks representative of those experienced in everyday life and relevant to the psychological process being investigated. This requires replacing laboratory stimuli by biotic and abiotic sounds recorded *in situ*—that is natural sounds shaped by the specific propagation characteristic of natural habitats (Mouterde et al., 2014)—and the identification of the repertoire of natural behaviors for humans via ethnographic studies aiming at characterizing “ordinary listening behaviors” in rural or wild settings. Evaluating the strength of rain or wind, composition and speed of running waters, discriminating dusk from night or more simply assessing changes in biodiversity in the surrounding acoustic environment may be important behaviors for people living in such places as they probably were for our ancestors. This enterprise belongs to cognitive ethology (Kingstone et al., 2008). Unfortunately, such studies are clearly lacking. To the best of our knowledge, at least two cognitive psychology studies suggest that sensory processing and attention may differ between rural and urban elderly people (Hirst et al., 2022). These studies indicate that rural environments are less complex than urban ones and situations typical of urban life such as road crossing require divided attention more than focused attention (Cassarino and Setti, 2016). More work is clearly warranted to characterize differences between urban, rural and wild settings, not only in terms of soundscape features (e.g., De Coensel et al., 2003) but also in terms of listening behaviors.

### 2.5 Summary

Taken together, these studies, summarized in Table 1, paint a complex picture and the available developmental data do not yet allow definitive conclusions to be drawn regarding the following competing hypotheses formulated to explain human ability to process natural sounds: (1) early sensitivity to water sounds suggests that infants and children perceive natural sounds through general auditory mechanisms distinct from those involved in speech processing and presumably shaped by ancestral selective pressures (Chen and Wiens, 2020; Lorenzi et al., 2023); (2) alternatively, newborns' similar preferences for monkey vocalizations and speech suggest that mechanisms involved in environmental sound and speech processing may initially develop together and then undergo specialization due to subsequent exposure (Perszyk and Waxman, 2016); (3) it is also possible that different classes of natural sounds are processed differently, depending on their acoustic characteristics or their survival value. Thus, primate or other animal vocalizations, which share some of their acoustic features with speech, such as harmonicity or slow temporal modulations, may be perceived differently from natural sounds and other texture-like sounds. Any attempt to test further these competing hypotheses should adopt an ecological perspective by capitalizing on the available databases of soundscapes collected in natural settings acoustically similar to human ancestral habitats and test basic auditory capacities presumably engaged in ordinary listening behaviors, some of which are currently being investigated in adults (Lorenzi et al., 2023).

**Table 1:** Summary of cited studies.

**Authors**	**Stimuli used**	**Type of measure**	**Summary of findings**
Ferry et al. (2013)	Blue-eyed Madagascar lemur vs. backward speech	Behavioral preference	Infants shift from a broad preference for both primate vocalization and speech to a preference for speech at 6 months
Perszyk and Waxman (2016)	Blue-eyed Madagascar lemur vs. backward speech	Behavioral preference	At 6 months infants categorize previously familiarized primate vocalization but not unfamiliar speech
Vouloumanos et al. (2010)	Rhesus macaque calls vs. nonsense speech sounds	Behavioral preference	Newborns show no preference for speech over rhesus vocalizations. 3-months-olds prefer speech to rhesus vocalizations
Shultz and Vouloumanos (2010)	Non-native speech, rhesus macaque calls, human non-communicative sounds, human communicative non-speech vocalizations, environmental sounds	Behavioral preference	Three-months-olds prefer speech over other stimuli
Minagawa-Kawai et al. (2011)	Native speech, non-native speech, emotional vocalizations, macaque calls, scrambled control sounds	fNIRS	In 4-months-olds primate vocalizations and speech activate similar brain regions. Speech elicits stronger activity on the left side, while monkey vocalizations elicits stronger activity on the right side
Lange-Küttner (2010)	Sea bird vs. garden bird songs	Behavioral preference	Five-seven-month-old infants prefer sea bird sounds. Adults prefer garden bird songs. This developmental change starts at 10-12 months. Early exposure helps shaping this bias
Santolin et al. (2019)	Bird song, forward and backward Mandarin Chinese	Eye-tracker	Four-month-old prefer bird songs over non-native speech
Gervain et al. (2014)	Scale-invariant vs. variable-scale synthetic water sounds	Behavioral preference	Five-month-old infants prefer scale-invariant water sounds
Gervain et al. (2016)	Scale-invariant vs. variable-scale synthetic water sounds	fNIRS	Newborns discriminate variable-scale and scale-invariant sounds in left frontal and temporal areas
Agrawal and Schachner (2023)	Hot vs. cold water sounds	Behavioral identification task	The ability to distinguish water sounds based on the temperature appears at 7–11 years of age
Martínez-Castilla et al. (2015)	Water sounds vs. walking sounds	Behavioral identification task	Children with Williams syndrome perform lower than their typically developing peers and similarly to those with Down syndrome in environmental sounds recognition

## 3 Perspectives and future directions

As we look ahead, the next crucial steps in the research agenda of the field of human auditory ecology involve a principled investigation of how natural soundscapes are perceived and processed across development into adulthood.

First, we need to explore children's basic perceptual sensitivities when processing natural sounds and soundscapes. Natural soundscapes show strong periodicity due to the day-night cycle, with distinct choruses at dawn and dusk forming a double-peaked circadian pattern of biological activity (Lorenzi et al., 2023), as well as due to the change of seasons. The biodiversity of a habitat also has its signature in its soundscape. Birds, insects, and amphibians, as primary contributors, produce vocalizations with faster temporal modulations, creating unique acoustic regularities that mammals, including human ancestors, have been exposed to for millions of years (Lorenzi et al., 2023). Exploring whether children, like adults, can discriminate between different habitats (e.g. savannah vs. rainforest), the same habitat at different times of the day or in different seasons—that is *global* attributes of natural auditory scenes (McMullin et al., 2024)—will provide comprehensive insights into how auditory discrimination develops and the role of experience in shaping this skill. Behavioral studies with adults (Apoux et al., 2023) suggest that exposure may not fundamentally impact discrimination of global attributes of natural soundscapes such as geolocation (habitat) or moment of the day. Several hours of training does not change adults' discrimination performance. May there be a critical period for attuning to the specifics of one's auditory environment just like there is a critical period for speech and music perception? Do young infants show greater or lesser sensitivity to natural sounds and soundscapes than adults? Can they discriminate all their relevant features? Do rural and urban children show similar sensitivities? Answering these questions is fundamental for a better understanding of human auditory ecology. Behavioral measures can be complemented by brain imaging techniques, now also readily applicable to even the youngest infants, to explore the neural correlates of these abilities. Indeed, integrating behavioral and neural measures could offer a more nuanced understanding. For example, infants might show neural signatures of being able to auditorily perceive the temperature of water, even if this sensitivity is not apparent behaviorally (Agrawal and Schachner, 2023).

Children's sensitivity to the characteristics of natural sounds and soundscapes can be built on in education and in raising awareness of ecology and biodiversity. By understanding children's sensitivity to biodiversity in soundscapes, we can develop educational strategies that nurture environmental consciousness in children. Indeed, an exciting aspect of this research is the potential to cultivate ecological awareness from an early age, ultimately fostering a deeper connection to and responsibility for the natural world.

Beyond basic auditory sensitivities, research shows that natural sounds, and in particular biodiversity (species richness and species abundance) and water sounds in natural soundscapes enhance wellbeing and exert restorative effects on their listeners (for a recent review, see Ratcliffe, 2021). Most of this research, however, have investigated restorative effects in adults. Much less is known about how natural sounds impact children's moods. Yet, these restorative effects may be useful in family, educational and even clinical contexts to improve children's moods, reduce stress and anxiety and promote wellbeing. More research is thus needed to understand how natural sounds or the lack of them impact children's psychological and mental health. This research could involve not only behavioral, but also physiological measures of mood such as using infant / child heart rate monitors to measure heart rate variability (HRV). Understanding these developmental trajectories will highlight the significance of natural soundscapes in enhancing cognitive and emotional health from infancy through adulthood.

This is also crucial for deaf and hard-of-hearing children. Currently, most intervention and rehabilitation programs (hearing aids, cochlear implants etc.) are optimized for speech perception and urban environments (Lorenzi et al., 2023). It is thus little known to what extent these programs and devices restore the objective and subjective percepts of natural sounds. It may thus be the case that deaf and hard-of-hearing people benefit less from natural sounds than their hearing peers because the hearing support or intervention they receive does not restore auditory experiences with natural sounds sufficiently well (Lorenzi et al., 2023; Miller-Viacava et al., 2023). More research is needed to improve natural soundscape perception and emotional responses through these devices, especially for early interventions.

## 4 Conclusions

In conclusion, understanding how children perceive natural sounds is crucial for unraveling the complexities of auditory development and its evolutionary underpinnings. Infants and young children are exposed to a rich tapestry of natural sounds from birth, and their perceptual abilities in this domain are only beginning to be understood. Current research highlights the importance of both biologically predisposed and experiential factors in shaping these auditory sensitivities. As we advance, adopting ecological approaches and utilizing natural soundscapes in experimental paradigms will provide deeper insights into the development of human auditory ecology. This will not only enhance our understanding of sensory processing but also inform strategies to harness the restorative and educational potential of natural sounds, ultimately promoting wellbeing and environmental awareness from an early age.
