# Supplementary material for: Developmental origins of natural sound perception
Source: Front Psychol. 2024 Dec 11;15:1474961. doi: 10.3389/fpsyg.2024.1474961 (PMC11669913; doi:10.3389/fpsyg.2024.1474961)
Supplement: Supplementary file 1 [file Data_Sheet_1.docx]

**Appendix**

The sound database and computational modelling used in the present study are described below. All sounds were equated in long-term root-mean-square (rms) power.

**1. Soundscape recordings (Figures 1 and 6)**

***1.1 Natural soundscapes***

MPS were computed for a **boreal forest** (Location: Algonquin Park; GPS: 45°13’25.41”N 78°35’25.31”W ; Altitude : 405m ; Time : 07:10am; Date: 24.03.2007), a **tropical forest** (Location: Sumatra, Ketambe ; GPS : 3°32’44.81”N 97°45’09.26”E ; Altitude : 338m ; Time : 06:25am ; Date : 09.03.1991), a **temperate forest** (Location: Nutter point, Twin lakes, Cheboygan, Michigan, USA; GPS: Latitude 45.7052895°, Longitude -84.7278262°; Altitude : 210m ; Time : 07:30am ; Date: 11.04.2012), a **desert** (Location: Gray Ranch, Chihuahuan desert; GPS : 31°27’38.62”N 108°51’35.33”W; Altitude: 1572m ; Time : 05:58am ; Date : 27.04.1992) and a **savannah** (Location: Munguezi, Zimbabwe, Africa; GPS: 20°58’04.65”S/32°19’14.58”E ; Altitude: 1468m ; Time : 04:22am; Date : 30.09.1996). Twelve to fifteen, 4-s long acoustic samples per type of soundscape. Sampling rate: 16 kHz. All sounds were used in Lorenzi et al. (2023). Source: B. Krause, Wild Sanctuary.

***1.2 Urban soundscapes***

MPS were computed for a **street with fast traffic** at midday (the soundscape is dominated by the sound of cars; estimated speed: 70 km/h) and **crowds** in indoor (e.g., restaurant, gatherings) environments. Street traffic: three to thirteen, 4-s long acoustic samples per category (restaurant, gathering, etc.). Source: S. Meunier, LMA, CNRS (Marseille, France). Crowds: twelve, 4-s long acoustic samples. Source: royalty free sound library Sound Jay (<https://www.soundjay.com/crowd-talking-1.html>), royalty free sound library SoundBible (<https://soundbible.com/2163-Party-Crowd.html>; <https://soundbible.com/1664-Restaurant-Ambiance.html>). Sampling rate for traffic and crowd recordings: 16 kHz.

**2. Natural and speech sounds (Figures 2 and 7)**

***2.1 Bird songs***

Eight bird species selected from the Risoux forest in Haut Jura (a protected European cold forest in the East of France). These species correspond to the most prevalent species of this habitat: *Erithacus rubecula* (European Robin), *Fringilla coelebs* (Common Chaffinch), *Periparus ater* (Coal Tit), *Phylloscopus collybita* (Common Chiffchaff), *Regulus regulus* (Goldcrest), *Sylvia atricapilla* (Eurasian Blackcap), *Turdus merula* (Common Blackbird), and *Turdus philomelos* (Song Thrush). For each species, fifteen to sixteen, 4-6 s long recordings selected from the library of Museum national d’Histoire naturelle (MNHN, https://sonotheque.mnhn.fr). Sampling rate: 16 kHz. Sources: J.-C. Roché & MNHN.

***2.2 Insects sounds***

Fifteen, 4-s long recordings of *Tettigonia viridissima*, the great green bush-cricket inhabiting the Risoux forest. Sampling rate: 44.1 kHz. Source: J. Sueur & MNHN.

***2.3 Primate vocalizations***

Fifteen, 0.6-1s long recordings. Sampling rate: 16 kHz. Source: Gemignani & Gervain (2024).

***2.4 Water sounds (boreal forest)***

Twelve, 4-s long recordings of a single headwater forest stream (Location: boreal Sweden (Övre Björntjärn: GPS: Latitude 64.126°, Longitude 18.776°; Date: 01 April – 31 October 2012–2015. The site was sampled several times to cover a large range of water temperature (0-17 °C) and discharge (16.2–113.8 L. s−1)). Sampling rate: 16 kHz. Source: Klaus et al. (2019).

***2.5 Speech sounds***

Corpus of 3-5-second-long sentences recorded in 10 different languages showing distinct rhythmic organizations from four female speakers: Dutch, English, French, Japanese, Polish, Spanish, Marathi, Turkish, Basque, and Zulu. Sixteen sentences per language. Sampling rate: 16 kHz. Source: Ramus et al. (1999). See also Varnet et al. (2017).

**3. Synthetic water sounds (Figure 4)**

Scale-invariant (“natural”) and scale-variable (“non natural”) synthetic water sounds (11-12 items of 18-sec-long samples per sound category). Sampling rate: 22 kHz. Sources: Geffen et al. (2011) and Gervain et al. (2014).

**4. Cold and hot water sounds (Figure 5)**

Cold (6-7,8°C) and hot water sounds (82-83,9°C) recorded in four pouring configurations (four, 4-5 s long samples per configuration: plastic, paper, porcelain, glass) for each temperature. Sampling rate: 48 kHz. Sources: Velasco et al., (2013); Agrawal and Schachner (2023).

**5. Auditory modelling**

All recordings of natural or synthetic water sounds were passed through a first bank of 35 gammatone bandpass filters tuned in the audio-frequency domain that simulated peripheral (cochlear) filtering. The center frequency of cochlear filters ranged between 50 and 9300 Hz. The center frequencies were spaced on an equivalent-rectangular-bandwidth (ERB) scale. Amplitude-modulation patterns (temporal envelopes) were then extracted at the output of each simulated cochlear filter and compressed. From these compressed temporal envelopes, marginal statistics (mean, variance, skewness and kurtosis) and cross-band correlations (i.e., the envelope correlation matrix) were estimated. The envelope mean provides a gross measure of the power spectrum (the so-called excitation pattern). The envelope variance, skew, and kurtosis statistics measure subband sparsity in each cochlear channel. For envelope skewness and kurtosis, signal sparsity reflects the presence of discrete and infrequent events. The envelope correlation matrix captures the linear relationship between pairs of envelope components and therefore estimates the coordination between temporal envelopes of distinct cochlear channels (for additional details, see [McWalter](https://www.ncbi.nlm.nih.gov/pubmed/?term=McWalter%20R%5BAuthor%5D&cauthor=true&cauthor_uid=28955191) & [Dau,](https://www.ncbi.nlm.nih.gov/pubmed/?term=Dau%20T%5BAuthor%5D&cauthor=true&cauthor_uid=28955191) 2017).

**6. Modulation Power Spectrum (MPS) and its statistics**

Detailed information about the computation of MPS and its statistics can be found in Singh & Theunissen, 2003. Following their work, we computed 7 statistics for each MPS, using their descriptive quantifiers of modulation spectra: Separability (**SE**), Asymmetry (**SY**), Low-pass coefficient (**LP**), Starriness (**ST**), and Modulation depth (**MD**), along with the slope of the power-law fit (*1/f^α^*) to the averaged temporal (**AT**) and spectral (**AS**) components of the modulation spectrum independently. These statistics are useful to summarize information about the structure of the MPS.

The MPS statistics can be interpreted as follows.

**SE**: assesses if the joint spectro-temporal modulations shown in the MPS can be predicted from spectral and temporal modulations independently. A value of 1 means totally predictable.

**SY**: compares the amount of up-sweeps and down-sweeps present in the spectrogram. A value of 0 means equal amount, positive for more down-sweeps, and negative for more up-sweeps.

**LP**: proportion of the total modulation power that is concentrated in a low-pass region (-10 to 10 Hz; 0 to 0,195 cycles/kHz).

**ST**: proportion of the total modulation power outside the low-pass region that is concentrated along the axes of the MPS. It highlights the modulations with either slow temporal and high spectral rates, or fast temporal and low spectral rates.

**MD**: proportion that contrasts the total modulation power at 0 rates vs all other rates.

**AT**: informs about the shape of the averaged temporal modulation spectrum for a range of frequencies between 3 and 100 Hz.

**AS**: informs about the shape of the averaged spectral modulation spectrum for a range of frequencies between 0.1 and 1 cycles/kHz.

**Figure captions**


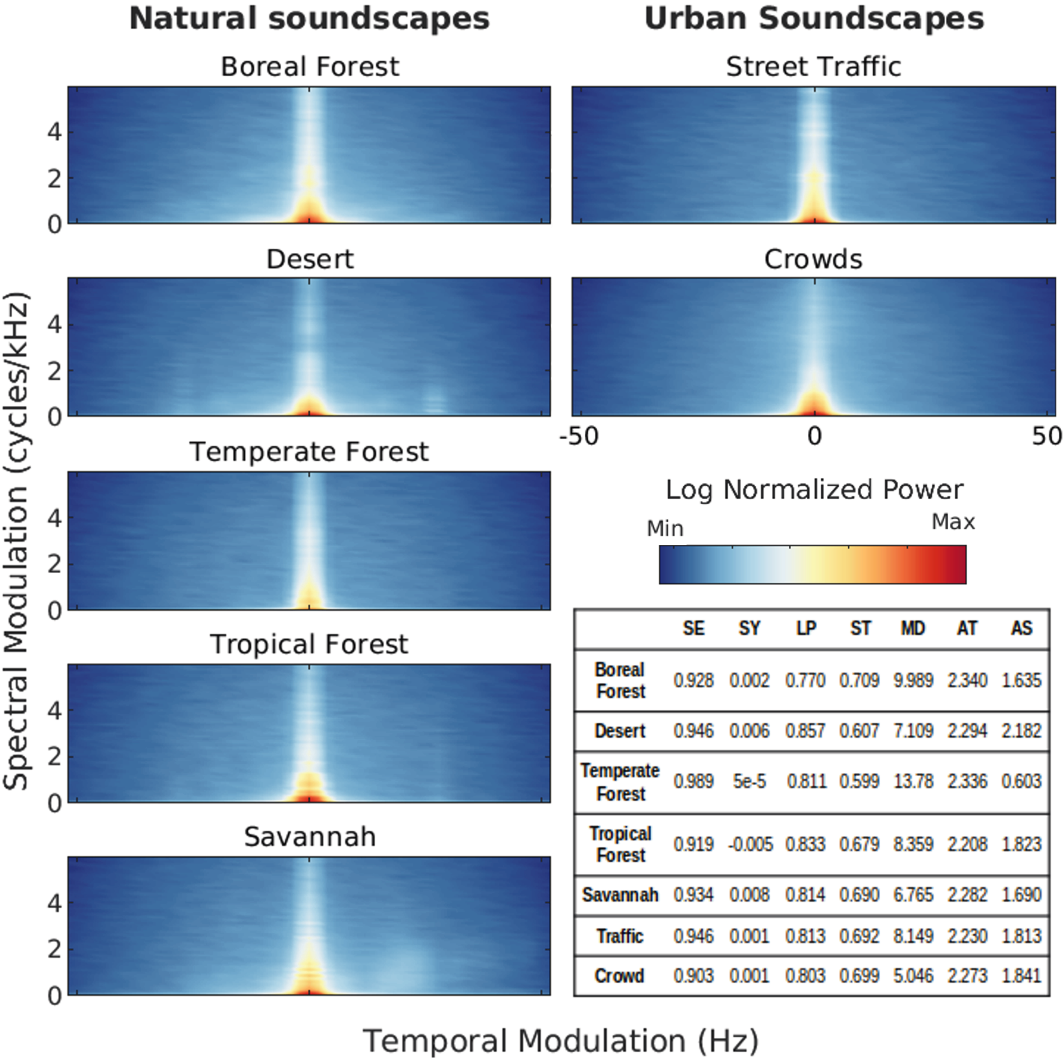


Figure 6: Average modulation power spectra (MPS) and modulation statistics of natural versus urban soundscapes. MPS were obtained by averaging the MPS computed for all recordings of the database for each sound category (e.g., boreal forest, street traffic, etc.). Modulation statistics computed from MPS: **SE**: Separability; **SY**: Asymmetry; **LP**: Low Pass coefficient; **ST**: Starriness; **MD**: Modulation Depth; **AT**: Slope of temporal-modulation spectrum (1/f^α^), **AS**: Slope of spectral modulation spectrum. The differences between modulation statistics in natural and urban soundscapes reflect the complex balance between biophony (the collective sound produced by human and non-human biological sources), geophony (the collective sound produced by geophysical sounds), anthropophony (the collective sound produced by man-made objects and machines) and sound-propagation characteristics (that differ strongly between indoor and outdoor settings, and between closed (forests) and open (savannah, desert) environments). See Figure 1 for further details.


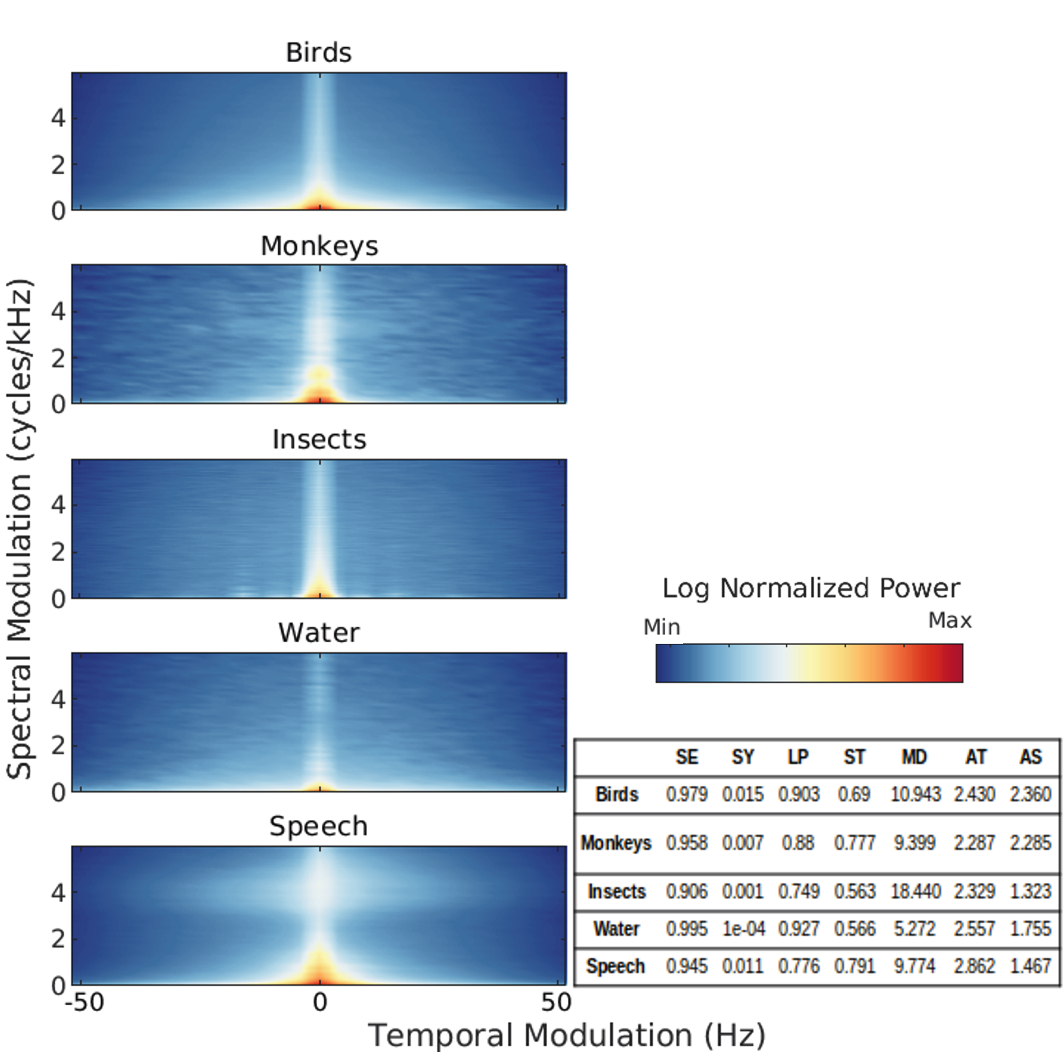


Figure 7: Average modulation power spectra (MPS) and modulation statistics of natural sounds (bird vocalizations, insect stridulations, primate vocalizations and water sounds) and speech sounds. MPS were obtained by averaging the MPS computed for all recordings of the database for each sound category (e.g., birds, sentences, etc.). See Figure 2 and 6 otherwise. Speech and non-human primate vocalizations show the highest levels of starriness whereas water and insect sounds show the lowest levels of starriness. Water sounds are more separable and show the lowest level of modulation depth than other natural and speech sounds, due to their noisy characteristics. All natural and speech sounds show a lowpass shape, except for insect sounds, presumably due to fast, periodic stridulations/timbalations. Insect sounds differ from all other sounds in terms of their spectral modulation spectrum.
